# P-160. Antibiotic Prescribing Trends and Outcomes in Outpatient Uncomplicated Diverticulitis: A Retrospective Analysis in Veterans Health Administration Between 2016 and 2023

**DOI:** 10.1093/ofid/ofaf695.384

**Published:** 2026-01-11

**Authors:** Shinya Hasegawa, Hiroyuki Suzuki

**Affiliations:** University of Iowa Carver College of Medicine, Iowa City, IA; University of Iowa Carver College of Medicine, Iowa City, IA

## Abstract

**Background:**

Diverticulitis is a common cause of emergency department (ED) visits for abdominal pain. Although recent guidelines recommend selective antibiotic use for uncomplicated cases, antibiotics remain commonly prescribed. Longitudinal trends in outpatient antibiotic use and outcomes of patients with uncomplicated diverticulitis are underreported.

**Figure:**
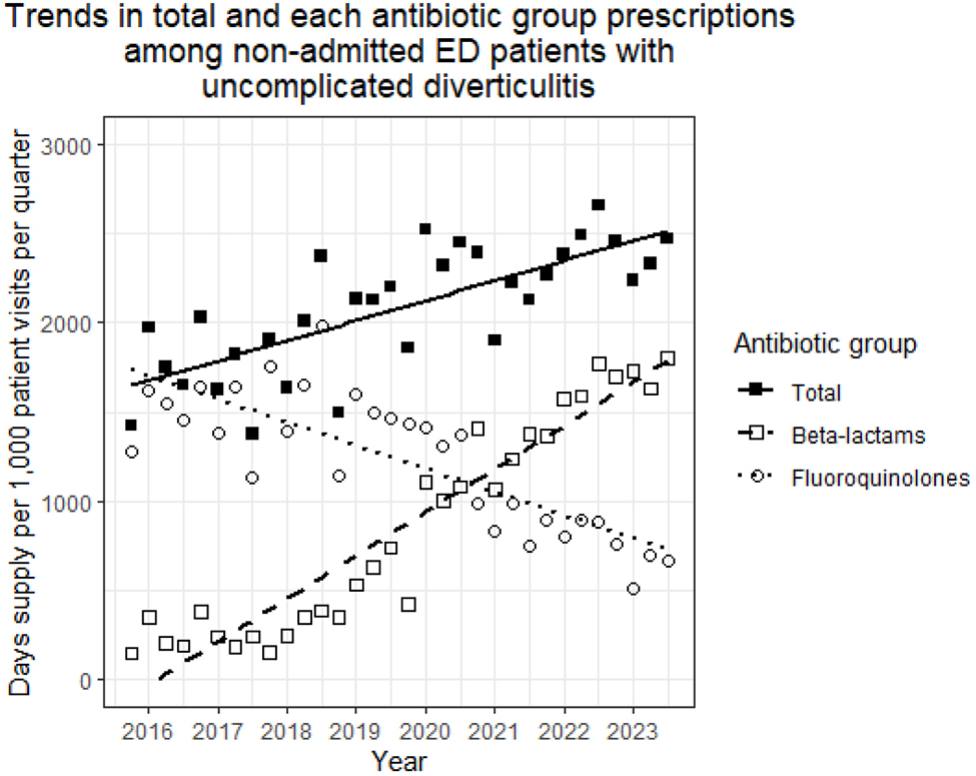
Trends in total and each antibiotic group prescriptions among non-admitted emergency department patients with uncomplicated diverticulitis. Abbreviations: ED, emergency department.

**Figure:**
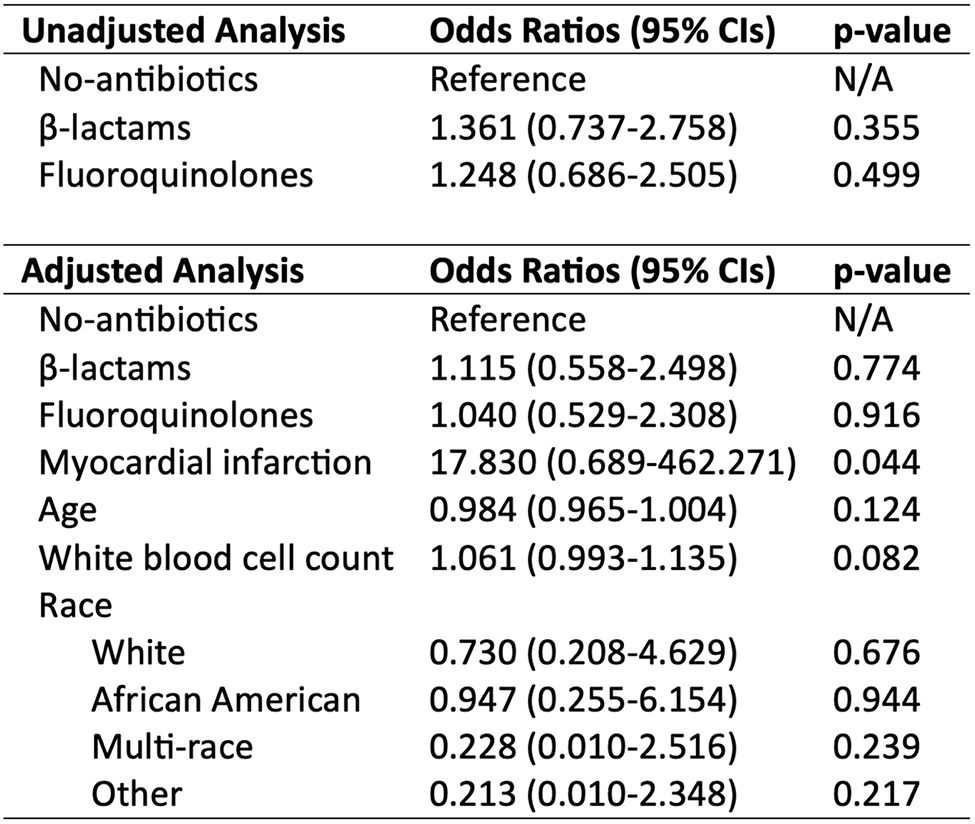
Logistic regression models to estimate odds ratios of 1-year hospital admission for uncomplicated diverticulitis Abbreviations: CI, confidence interval.

**Methods:**

We conducted a retrospective cohort study (2016–2023) using the Veterans Health Administration Corporate Data Warehouse to identify ED visits for a first episode of diverticulitis. Patients admitted to the hospital within 48 hours of the ED visit were excluded. We identified uncomplicated cases using International Classification Disease-10 codes of diverticulitis without perforation or abscess, Charlson Comorbidity Index < 2, white blood cell count < 15 x 10^3 /μL, and C-reactive protein < 14 mg/dL. Patients were categorized into three groups based on the type of antibiotics prescribed; β-lactams, fluoroquinolones, or no-antibiotics. Linear regression was used to assess time trends in antibiotic treatment. The primary outcome was 1-year hospital admission for diverticulitis, assessed using multivariable generalized linear modeling, including variables with univariate p-value < .20.

**Results:**

Among 12,197 patients (mean age 60 years; 88.9% male) from 77 hospitals, 3,008 (24.7%) had uncomplicated diverticulitis. Of these, only 215 (7.9%) received no antibiotics, while 1,024 (34.0%) and 1,491 (49.6%) were classified into the β-lactams and fluoroquinolones groups, respectively. The median antibiotic duration was 10 days (interquartile range, 7-10 days). Antibiotic prescriptions among uncomplicated cases increased over time, with a shift from fluoroquinolone to β-lactam use (all p < .001) (Figure). In multivariable analysis, neither β-lactams nor fluoroquinolones use was significantly associated with 1-year hospital admission (Table).

**Conclusion:**

Antibiotic prescriptions for uncomplicated diverticulitis increased over time, with a shift toward increased β-lactam use. Many antibiotics may be prescribed unnecessarily in this population. Further studies are needed to better define low-risk patients and develop strategies to reduce unnecessary antibiotic use.

**Disclosures:**

All Authors: No reported disclosures

